# EPI-X4, a novel endogenous antagonist of CXCR4

**DOI:** 10.18632/oncotarget.6037

**Published:** 2015-10-08

**Authors:** Christian Buske, Frank Kirchhoff, Jan Münch

**Affiliations:** Ulm University Medical Center, Institute of Molecular Virology, Ulm, Germany

**Keywords:** CXCR4, EPI-X4

Activation of CXC-motif-chemokine receptor 4 (CXCR4) signaling by the chemokine CXCL12 (also known as SDF-1α) plays a key role in many physiological processes including organogenesis, hematopoiesis, and immune responses. Consequently, CXCR4 or CXCL12 knockouts in mice are embryonic lethal because of impaired hematopoiesis, defects in organ development, and vascularization. Deregulation of CXCR4-CXCL12 signaling in humans is involved in multiple diseases, such as various forms of cancers, rheumatoid arthritis, autoimmune disorders, inflammation and cardiovascular diseases. Finally, CXCR4 serves as a major co-receptor for HIV-1 entry. Thus, there is a great interest to understand the CXCR4-CXCL12 crosstalk and to develop clinically applicable CXCR4 ligands, which antagonize aberrant receptor activity. So far, research has focused on man-made synthetic CXCR4 antagonists.

Zirafi *et al* recently reported the discovery of a novel endogenous ligand of CXCR4, demonstrating that CXCR4 activity can be modulated by a naturally occurring ligand [1]. By screening a human hemofiltrate-derived peptide library [2], they identified a 16-mer fragment of human serum albumin - the most abundant protein in plasma - as potent inhibitor of CXCR4-tropic HIV-1 strains. They termed this peptide EPI-X4 (Endogenous Peptide Inhibitor of CXCR4) and demonstrated that it binds to the second extracellular loop of CXCR4 to prevent engagement of the viral gp120 protein and consequently HIV-1 entry. Binding of EPI-X4 to CXCR4 was highly specific and suppressed both, basal and CXCL12-induced signaling. Furthermore, this endogenous CXCR4 antagonist blocked CXCL12-mediated receptor internalization and suppressed the migration and invasion of cancer cells towards a CXCL12 gradient, suggesting that EPI-X4 may have anti-metastatic activity. Studies in mice suggest that EPI-X4 has therapeutic potential as the peptide mobilized hematopoietic stem/progenitor cells and inhibited recruitment of inflammatory immune cells into the lung in an asthma model.

The study demonstrates that EPI-X4 is generated from the abundant albumin precursor by aspartic proteases, such as Cathepsin D and E [1]. These proteases are available in plasma but mainly found in lysosomes and in specialized secretory granules of immune cells, such as neutrophils or cytotoxic T cells. They are activated under acidic conditions and acidification of human plasma was sufficient to generate bioactive concentrations of EPI-X4. The albumin precursor is abundant in the vascular and extravascular space and the EPI-X4 releasing enzymes are ubiquitously expressed. Thus, the prerequisites for the generation of this endogenous CXCR4 antagonist are given almost everywhere in the human body. Acidic pH conditions are characteristic for inflammatory and tumor tissues, and local acidification is emerging as key regulatory mechanism of innate immunity [4]. Thus, EPI-X4 might be specifically generated at sites of inflammation and immune activity to down-modulate local CXCR4-mediated responses, such as cellular migration or proliferation. Its activity is tightly regulated since EPI-X4 has a plasma half-life of only ∼17 minutes and is not detectable in the circulation of healthy individuals [1, 3]. Altogether, the data of Zirafi and colleagues suggest that EPI-X4 may play a role in homeostasis, immune defense and inflammation.

Dysregulation of CXCR4 is involved in various diseases, including tumor proliferation or dissemination. For example, increased CXCR4 expression is observed in many types of cancer and promotes invasion and proliferation of tumor cells as well as tumor-associated neoangiogenesis. Moreover, CXCL12 expression levels are elevated at metastatic sites and responsible for dissemination of malignant cells. In addition, activating mutations of CXCR4 are detectable in around 30 % of patients with Waldenström's Macroglobulinemia and are associated with a significant inferior response to the BTK inhibitor ibrutinib [5]. Two independent studies also demonstrated that CXCR4 is critical for T cell acute lymphoblastic leukemia (T-ALL) development and that CXCR4 antagonism suppresses T-ALL growths [6, 7]. Thus, CXCR4 is an important drug target and several synthetic antagonists are currently evaluated in preclinical and clinical studies.

To date, however, only Mozobiol® (AMD3100) has been approved for clinical use. Since AMD3100 causes significant side effects, it is only used for the mobilization of hematopoietic stem cells in combination with G-CSF for collection and subsequent transplantation in patients with non-Hodgkin lymphoma and myeloma, but is not suitable for the treatment of chronic CXCR4-linked disease. In contrast to AMD3100, EPI-X4 also reduces basal CXCR4 signaling in the absence of CXCL12 and does not interact with CXCR7, whereas AMD3100 acts as allosteric agonist of this receptor [1]. Notably, some synthetic derivatives of EPI-X4 showed greatly increased plasma stability and blocked CXCR4 signaling more efficiently and specifically than AMD3100 [1]. Thus, EPI-X4 has interesting features for clinical development and further studies on its therapeutic potential are highly warranted.
